# Successful diagnosis and treatment of pulmonary aspergillosis-related malignant catatonia using propofol and quetiapine: A case report

**DOI:** 10.1097/MD.0000000000025967

**Published:** 2021-05-14

**Authors:** Kazuhito Nomura, Sonoko Sakawaki, Eiji Sakawaki, Ayumu Yamaoka, Wakiko Aisaka, Hiroyuki Okamoto, Yoshihiro Takeyama, Shuji Uemura, Eichi Narimatsu

**Affiliations:** a Department of Emergency Medicine, Sapporo Medical University Hospital, Sapporo-shi; b Department of Emergency Medicine, Hakodate Municipal Hospital, Hakodate-shi; c Department of Neurosurgery, Sunagawa City Medical Center, Sunagawa-shi; d Department of Emergency Medicine, Teine Keijinkai Hospital, Sapporo-shi, Hokkaido, Japan.

**Keywords:** deep-seated mycosis, diagnostic test, malignant catatonia, pharmacology, propofol, pulmonary aspergillosis, quetiapine

## Abstract

**Introduction:**

Malignant catatonia (MC) is a movement disorder syndrome characterized by immobility, rigidity, and consciousness disorders that develops in association with mental and physical diseases. It is often fatal due to hyperthermia, rhabdomyolysis, and acute kidney injury. Its clinical symptoms are similar to those of another disorder, neuroleptic malignant syndrome (NMS), and it is often difficult to distinguish between the 2 disorders.

**Patient concerns:**

An Asian woman in her 60s with history of schizophrenia. She was admitted to our hospital because of symptoms such as fever, unconsciousness, and muscle rigidity. Blood tests showed kidney injury and high creatinine kinase levels.

**Diagnoses:**

At the time of admission, she had been diagnosed with NMS complicated by pulmonary aspergillosis and was undergoing treatment although there was no improvement.

**Interventions:**

Subsequently, the administration of propofol, a gamma-aminobutyric acid A agonist, markedly improved the symptoms, and the diagnosis was corrected to MC. At the beginning of her hospitalization, she received dantrolene, bromocriptine, amantadine, and L-3,4-dihydroxyphenylalanine as treatment for NMS, but her symptoms did not improve. With propofol, which is used for sedation, her catatonic symptoms improved markedly. Quetiapine administration further improved the symptoms, and it eventually resolved completely.

**Outcomes:**

The patient's MC was in remission. Prolonged intensive care management resulted in a decline in activities of daily living, and she required rehabilitation at another hospital.

**Conclusion:**

This is the first report of MC with suspected involvement of pulmonary aspergillosis. MC differs from NMS, in that it is treated more effectively with gamma-aminobutyric acid A agonists. Although benzodiazepines are the first choice for the diagnosis and treatment of MC, they are ineffective for majority of patients with schizophrenia. However, even in such cases, propofol and quetiapine are effective, and they facilitate diagnosis and treatment.

## 1 Introduction

Both neuroleptic malignant syndrome (NMS) and malignant catatonia (MC) are dyskinetic syndromes characterized by abnormalities such as high fever, muscle rigidity, and altered consciousness.^[1]^ Cases of MC were reported before the development of antipsychotic drugs, and this type of catatonia can advance regardless of the administration of such drugs. In contrast, NMS differs in that it has always been associated with the administration of antipsychotic drugs. However, it is not uncommon for patients receiving antipsychotic drugs to develop MC, as has been reported previously.^[2]^ Since they exhibit similar symptoms and there is no disease-specific test, it is extremely difficult to distinguish MC from NMS, and treatment is often difficult without a correct diagnosis.^[3]^ In this report, we discuss the case of a patient who was initially diagnosed with refractory NMS complicated by deep *Aspergillus* infection, but in whom the diagnosis was subsequently corrected to MC due to the unexpected positive effect of propofol followed by quetiapine.

## 2 Case presentation

**Figure 1:**
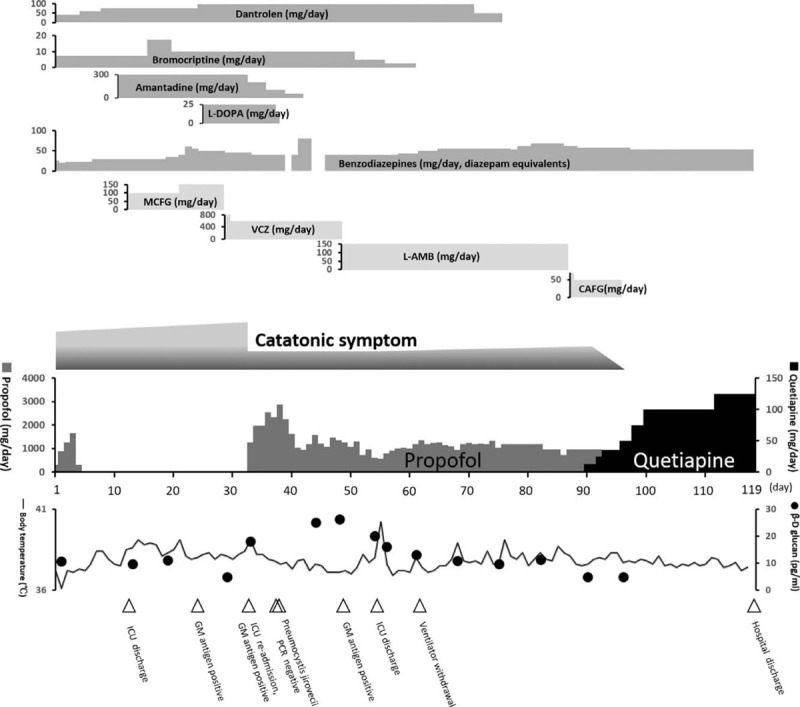
The time course from admission to discharge. CAFG = caspofungin, GM = galactomannan, ICU = intensive care unit, L-AMB = liposomal amphotericin B, MCFG = micafungin, VCZ = voriconazole.

The patient was an Asian woman in her 60s. She had a history of schizophrenia and cholelithiasis Fig. 1. She received brotizolam 0.25 mg/d, flunitrazepam 4 mg/d, quetiapine 600 mg/d, olanzapine 20 mg/d, powdered rhubarb (a type of Japanese herbal laxative) 9 g/d, magnesium oxide 9 g/d, rebamipide 300 mg/d, and bromazepam 15 mg/d. There was no change in the type or amount of drugs taken within the 6 months before her admission to our hospital. During the summer, she was transferred to a local hospital because she had a fever of 40°C and exhibited symptoms of malaise. She was hospitalized with a diagnosis of suspected urinary tract infection because she had a slightly elevated white blood cell count in her urine. Her blood test on the day after hospitalization revealed abnormalities in serum creatine kinase (154,000 IU/L) and serum creatinine (4.3 mg/dL). The attending physician diagnosed her with NMS and acute kidney injury, following which she was referred to our hospital for treatment.

**Figure 2:**
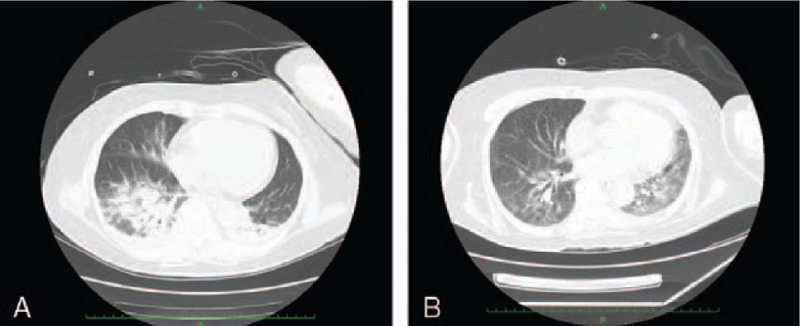
CT findings. (A) Lung CT on day 1. An infiltrative shadow due to pneumonia can be observed spreading to both lung fields. (B) Lung CT on day 84. An inflammatory ground glass opacity can be observed extending into the left lung field. CT = computed tomography.

On day 1, she was admitted to the intensive care unit (ICU). Her level of consciousness was Glasgow Coma Scale E3V4M6, and she was able to respond to simple commands. Strong rigidity was observed in the upper and lower limbs. Other vital signs were as follows: blood pressure, 130/84 mm Hg; heart rate, 92 bpm; respiratory rate, 15/min; percutaneous oxygen saturation: 100% (O_2_ administered at 5 L/min); and body temperature, 38.5°C (axillary temperature). She was diagnosed with NMS because she exhibited the major symptoms outlined in Levenson diagnostic criteria,^[4]^ including hyperthermia, muscle rigidity, elevation of serum creatine kinase, as well as changes in the state of consciousness. Initially, we administered dantrolene (40 mg/d) and bromocriptine (7.5 mg/d). Given the presence of infiltrative shadows in both lungs on computed tomography (CT) (Fig. 2A), her poor general condition, and her consciousness disorder, we also performed endotracheal intubation and mechanical ventilation, intravenous rehydration, cooling, and continuous renal replacement therapy. Head and abdominal CT examinations did not reveal any findings related to the patient's symptoms.

On day 5, her blood tests and respiratory function improved, following which she was extubated and mechanical ventilation was terminated. On day 6, dantrolene was increased to 60 mg/d because her rigidity and hyperthermia had not improved. Treatment with diazepam (5 mg/d) and flunitrazepam (5 mg/d) was initiated for her prominent insomnia.

On day 13, she left the ICU because her renal function had improved, and she no longer needed blood purification. On day 14, she was diagnosed with pulmonary mycosis due to the infiltrative shadow observed on CT on day 1 (Fig. 2A) and an elevated β-D glucan level from her blood sampling on day 2. The delay in diagnosis occurred because the β-D glucan test in our hospital requires 1 to 2 weeks before results are available. We began to give her micafungin (MCFG) for the treatment of pulmonary mycosis.

From day 16 to day 24, since her rigidity and hyperthermia had not improved, the doses of medications were increased as follows: MCFG to 150 mg/d, dantrolene to 100 mg/d, and bromocriptine to 17.5 mg/d. Amantadine (300 mg/d) and L-3,4-dihydroxyphenylalanine (L-DOPA) (25 mg/d) were also added.

On day 29, the patient's day 23 β-D glucan level had increased to 11.43 pg/mL, and her *Aspergillus* antigen test was also positive. She was thus diagnosed with a deep-seated *Aspergillus* infection. We considered MCFG to be ineffective and changed the antifungal drug to voriconazole (800 mg/d, lowered to 600 mg/d on the following day).

On day 34, she became restless due to extreme excitement and was unable to respond to directions. She was unable to communicate, shouted incomprehensibly, and refused to undergo medical treatment. She exhibited continuous movement in bed and could no longer rest. She would often stare diagonally upward with what appeared to be a startled expression on her face, although she did not make eye contact with the medical staff. Benzodiazepines were ineffective for her symptoms, and her respiratory status further worsened with decreased percutaneous oxygen saturation. We determined that she was in septic shock and delirium due to exacerbation of a deep-seated fungal infection. Consequently, we transferred her to the ICU and again performed tracheal intubation and mechanical ventilation. Intravenous administration of 100 mg of propofol for sedation during tracheal intubation immediately relieved her muscle rigidity, and she became able to comply with instructions. Before propofol administration, the patient's Bush-Francis Catatonia Rating Scale score^[5]^ was 25 points (Table 1). Her diagnosis was revised from NMS to MC because she met the 4 criteria for the diagnosis of MC (severe catatonia symptoms, hyperthermia, hemodynamic instability, and increased muscle rigidity)^[6,7]^ and because she did not respond to NMS treatment. Since dantrolene, bromocriptine, amantadine, and L-DOPA have side effects when the administration is stopped abruptly, we decided to discontinue administration after gradually decreasing the dose.

**Table 1:** The patient's Bush-Francis Catatonia Rating Scale scores on day 34 (before propofol administration).

Excitement	+3
Immobility/stupor	0
Mutism	0
Staring	+1
Posturing/catalepsy	0
Grimacing	+3
Echopraxia/echolalia	0
Stereotypy	0
Mannerisms	0
Verbigeration	+3
Rigidity	+3
Negativism	+3
Waxy flexibility	0
Withdrawal	+3
Impulsivity	0
Automatic obedience	0
Mitgehen	0
Gegenhalten	0
Ambitendency	0
Grasp reflex	0
Perseveration	+3
Combativeness	0
Autonomic abnormality	+3

On day 46, her β-D glucan results remained positive. We judged voriconazole to be ineffective and changed the antifungal drug to liposomal amphotericin B (L-AMB) (150 mg/d).

On day 86, we replaced L-AMB with caspofungin (70 mg/d) (50 mg/d from the following day) because her β-D glucan results remained positive despite prolonged administration of L-AMB. In addition, we observed a ground glass opacity in the left lung (Fig. 2B).

On day 91, we initiated treatment with quetiapine at 12.5 mg/d because propofol treatment alone did not result in amelioration of symptoms. Its effects had in fact diminished, and consent modified electroconvulsive therapy could not be obtained from the patient and her relatives. Administration of quetiapine did not cause exacerbation of hyperthermia or muscle rigidity.

On day 93, the β-D glucan level became negative. On day 95, the patient's consciousness was Glasgow Coma Scale E4V5M6. She was able to participate in a conversation through the use of a tracheostomy tube with a speaking valve. Her muscle rigidity had been nearly eliminated, and since we determined that the patient's MC was almost in remission, administration of propofol was terminated. On day 96, the β-D glucan remained negative, and her C-reactive protein level decreased. Thus, we concluded that the deep-seated aspergillosis had been cured, and administration of caspofungin was terminated.

On day 119, the patient's general condition and consciousness were stable. There were no abnormalities in consciousness or behavior reminiscent of excitement, stupor, or negativism. She was left with a walking disability and skilled finger movement disorder due to long-term bed rest and was transferred to another hospital for rehabilitation and treatment for schizophrenia.

## 3 Discussion

The present report is the first to suggest an association between pulmonary aspergillosis and the development of MC. Recent studies have reported that MC develops in combination with various inflammatory diseases such as infections and autoimmune diseases.^[8]^ Our patient was diagnosed with pulmonary aspergillosis based on a pulmonary CT image of pneumonia, elevated β-D glucan levels in her blood, and positive findings from an *Aspergillus* antigen test. As the duration of illness was nearly identical for her MC and deep-seated aspergillosis, we strongly suspect that these diseases were associated.

In our patient, we observed no inflammatory disease outside the lungs, including meningitis and sinusitis. She did not meet the diagnostic criteria outlined by the European Organization for Research and Treatment of Cancer Mycoses Study Group Criteria.^[9]^ However, this is not uncommon in cases of invasive aspergillosis in patients with uncomplicated hematologic neoplastic disease.^[9]^

Although samples (sputum, urine, blood) were collected several times, fungal culture tests were not successful in our patient, and failure to conduct antifungal susceptibility tests made the selection of the best antifungal drug difficult. However, enzyme-linked immunosorbent assays for the *Aspergillus* galactomannan (GM) antigen (enzyme-linked immunosorbent assay, PLATELIA ASPERGILLUS Ag) were strongly positive on days 23, 34, 49. In contrast, *Candida* antigen tests (latex agglutination reaction, CAND-TEC, LSI Medience, Tokyo, Japan) on these days were negative. Furthermore, polymerase chain reaction analyses for *Pneumocystis jirovecii* on day 37 (sputum) and day 38 (bronchoalveolar lavage fluid) were negative. Conditions that can lead to false-positive GM antigens include the use of some penicillins and the establishment of GM-producing microorganisms.^[9]^

Our patient's β-D glucan level was measured using a β-glucan test (Waco Pure Chemical Industries, Osaka, Japan, cutoff value: 11 pg/mL). β-D glucan can increase due to the administration of drugs containing β-D glucan, renal replacement therapy using cellulose membranes, and sepsis caused by *Alcaligenes faecalis*.^[10,11]^ However, none of these were observed in our patient.

The patient had no concomitant immunodeficiency diseases, including hematological malignancies, and no history of immunosuppressive drug use. This patient was not tested for human immunodeficiency virus (HIV), as there was no drop in lymphocyte count, and she had no history of repeated infections. Since the prevalence of HIV in Japan is low, and the patient had no history of sexual contact with an unspecified number of people, we concluded that it was extremely unlikely that the patient had HIV infection. However, her aspergillosis was refractory and required multiple antifungal drug changes. In recent years, the development of voriconazole resistance in *Aspergillus* has become a worldwide problem, and 1 multicenter study reported that approximately 20% of patients with pulmonary aspergillosis were infected with voriconazole-resistant strains.^[12]^

Previous studies have also reported that MCFG and L-AMB are only about 60% and 40% effective against *Aspergillus* species, respectively.^[13,14]^ The *Aspergillus* strain in this case may have been such a strain with low drug sensitivity.

In addition to MC and NMS, delirium due to invasive *Aspergillus* infection represents a differential cause of psychiatric symptoms in our patient. However, this is unlikely because propofol administration, a risk factor for delirium,^[15,16]^ greatly improved her psychiatric symptoms and involuntary movements (eg, muscle rigidity).

As in our case, patients with schizophrenia receiving antipsychotics often develop symptoms of catatonia. A common link in many of these cases is that determining correct treatment can be challenging due to the difficulty in distinguishing MC from NMS.^[17,18]^ Recent studies have revealed that gamma-aminobutyric acid A (GABA_A_) receptor activity is decreased in the lateral orbital cortex of patients with catatonia, which is thought to be deeply involved in the mechanism of catatonia development. It is well known that the GABA_A_ agonist benzodiazepine is highly effective against MC, but it does not work well against NMS.^[19,20]^ We strongly suspected MC rather than NMS because the GABA_A_ agonist propofol was very effective in this case, while dopamine agonists – which are used successfully against NMS – were ineffective. However, our patient did not respond to benzodiazepines, which may be because benzodiazepines are only 20% to 30% effective in patients with schizophrenic catatonia.^[19]^

Two studies have reported unintentional improvement of catatonia symptoms when propofol was used for sedation in patients with catatonia.^[21,22]^ Hyperactivity of N-methyl-D-aspartate receptors is also considered among the causes leading to the development of MC.^[8]^ Propofol not only acts as a GABA_A_ receptor agonist but also expresses antagonistic action at N-methyl-D-aspartate receptors,^[23]^ which may explain its significant effects in this case. Therefore, to aid in diagnosis and treatment, we propose that a propofol challenge test be performed in patients with catatonia symptoms who have not responded to NMS treatment and benzodiazepine administration.

In our case, remission was not achieved using propofol alone. Addition of quetiapine finally eliminated the catatonia symptoms. One study has suggested that, although catatonia is more likely to occur in patients with schizophrenia, it is a different condition than schizophrenia itself, in which antipsychotics are ineffective.^[24]^ Certainly, first-generation antipsychotics are not recommended for use in patients with catatonia due to the high risk of developing NMS.^[3,4]^ However, several reports have indicated that second-generation antipsychotics such as quetiapine can be successful.^[20,25]^

Quetiapine acts as GABA_A_ receptor agonist. In our case, we considered the GABA_A_ agonistic effect of quetiapine to be associated with remission of MC. Separately, the remission of MC coincided with the resolution of refractory deep-seated aspergillosis. It is possible that recovery from the inflammatory condition further facilitated the remission of MC.

In summary, the present case is the first to show that various inflammatory diseases are involved in the development of MC, and that deep-seated aspergillosis is among them. Our findings suggest that, when a patient with schizophrenia treated with an antipsychotic drug or benzodiazepine develops MC, the GABA_A_ agonist propofol can aid in diagnosis and treatment.

## Acknowledgments

The authors would like to express their sincere appreciation and gratitude to the nurses for their dedicated care of this patient.

## Author contributions

**Conceptualization:** Kazuhito Nomura.

**Data curation:** Kazuhito Nomura, Sonoko Sakawaki.

**Investigation:** Kazuhito Nomura, Sonoko Sakawaki.

**Supervision:** Sonoko Sakawaki, Eiji Sakawaki, Ayumu Yamaoka, Wakiko Aisaka, Hiroyuki Okamoto, Yoshihiro Takeyama, Shuji Uemura, Eichi Narimatsu.

**Writing – original draft:** Kazuhito Nomura.

## References

[1] Johnson RA. NMS, and why we should call it (malignant) catatonia. J J P 2006;20: Article 1.

[2] Chiou YJ Lee Y Lin CC Huang TL. A case report of catatonia and neuroleptic malignant syndrome with multiple treatment modalities: short communication and literature review. Medicine 2015;94:e1752. 26512569 10.1097/MD.0000000000001752 PMC4985383

[3] Caroff SN Mann SC Keck PE. Specific treatment of the neuroleptic malignant syndrome. Biol Psychiatry 1998;44:378–81. 9777166 10.1016/s0006-3223(97)00529-5

[4] Levenson JL. Neuroleptic malignant syndrome. Am J Psychiatry 1985;142:1137–45. 2863986 10.1176/ajp.142.10.1137

[5] Bush G Fink M Petrides G Dowling F Francis A. Catatonia. I. Rating scale and standardized examination. Acta Psychiatr Scand 1996;93:129–36. 8686483 10.1111/j.1600-0447.1996.tb09814.x

[6] Häfner H Kasper S. Acute life-threatening catatonia. Nervenarzt 1982;53:385–94. 6126826

[7] Philbrick KL Rummans TA. Malignant catatonia. J Neuropsychiatry Clin Neurosci 1994;6:1–3. 7908547 10.1176/jnp.6.1.1

[8] Rogers JP Pollak TA Blackman G David AS. Catatonia and the immune system: a review. Lancet Psychiatry 2019;6:620–30. 31196793 10.1016/S2215-0366(19)30190-7 PMC7185541

[9] De Pauw B Walsh TJ Donnelly JP. Revised definitions of invasive fungal disease from the European Organization for Research and Treatment of Cancer/Invasive Fungal Infections Cooperative Group and the National Institute of Allergy and Infectious Diseases Mycoses Study Group (EORTC/MSG) consensus group. Clin Infect Dis 2008;46:1813–21. 18462102 10.1086/588660 PMC2671227

[10] Verweij PE Mennink-Kersten MASH. Issues with galactomannan testing. Med Mycol 2006;44:S179–83. 30408901 10.1080/13693780600904918

[11] Wright WF Overman SB Ribes JA. (1-3)-β-D-glucan assay: a review of its laboratory and clinical application. Lab Med 2011;42:679–85.

[12] Lestrade PP Bentvelsen RG Schauwvlieghe AFAD. Voriconazole resistance and mortality in invasive aspergillosis: a multicenter retrospective cohort study. Clin Infect Dis 2019;68:1463–71. 30307492 10.1093/cid/ciy859

[13] Kohno S Masaoka T Yamaguchi H. A multicenter, open-label clinical study of micafungin (FK463) in the treatment of deep-seated mycosis in Japan. Scand J Infect Dis 2004;36:372–9. 15287383 10.1080/00365540410020406

[14] Walsh TJ Hiemenz JW Seibel NL. Amphotericin B lipid complex for invasive fungal infections: analysis of safety and efficacy in 556 cases. Clin Infect Dis 1998;26:1383–96. 9636868 10.1086/516353

[15] Pandharipande P Cotton BA Shintani A. Prevalence and risk factors for development of delirium in surgical and trauma ICU patients. J Trauma 2008;65:34–41. 18580517 10.1097/TA.0b013e31814b2c4d PMC3773485

[16] Pandharipande PP Pun BT Herr DL. Effect of sedation with dexmedetomidine vs lorazepam on acute brain dysfunction in mechanically ventilated patients: the MENDS randomized controlled trial. JAMA 2007;298:2644–53. 18073360 10.1001/jama.298.22.2644

[17] Komatsu T Nomura T Takami H. Catatonic symptoms appearing before autonomic symptoms help distinguish neuroleptic malignant syndrome from malignant catatonia. Intern Med 2016;55:2893–7. 27725556 10.2169/internalmedicine.55.6613 PMC5088557

[18] Fukai M Hirosawa T Takahashi T Kaneda R Kikuchi M Minabe Y. Clonazepam improves dopamine supersensitivity in a schizophrenia patient: a case report. Ther Adv Psychopharmacol 2017;7:113–7. 28348731 10.1177/2045125316681750 PMC5354128

[19] Rosebush PI Mazurek MF. Caroff SN Mann SC Francis A Fricchione G. Pharmacotherapy. Catatonia: From Psychopathology to Neurobiology. Washington DC: American Psychiatric Publishing; 2004. 141–50.

[20] Pelzer AC van der Heijden FM den Boer E. Systematic review of catatonia treatment. Neuropsychiatr Dis Treat 2018;14:317–26. 29398916 10.2147/NDT.S147897 PMC5775747

[21] Alfson ED Awosika OO Singhal T Fricchione GL. Lysis of catatonic withdrawal by propofol in a bone-marrow transplant recipient with adenovirus limbic encephalitis. Psychosomatics 2013;54:192–5. 22705183 10.1016/j.psym.2012.03.003

[22] Heekin RD Bradshaw K Calarge CA. First known case of catatonia due to cyclosporine A-related neurotoxicity in a pediatric patient with steroid-resistant nephrotic syndrome. BMC Psychiatry 2019;19:123. 31014303 10.1186/s12888-019-2107-6 PMC6480487

[23] Orser BA Bertlik M Wang LY MacDonald JF. Inhibition by propofol (2,6 di-isopropylphenol) of the N-methyl-D-aspartate subtype of glutamate receptor in cultured hippocampal neurones. Br J Pharmacol 1995;116:1761–8. 8528557 10.1111/j.1476-5381.1995.tb16660.x PMC1909100

[24] Ohi K Kuwata A Shimada T. Response to benzodiazepines and the clinical course in malignant catatonia associated with schizophrenia: a case report. Medicine 2017;96:e6566. 28422845 10.1097/MD.0000000000006566 PMC5406061

[25] Yoshimura B Hirota T Takaki M Kishi Y. Is quetiapine suitable for treatment of acute schizophrenia with catatonic stupor?. A case series of 39 patients. Neuropsychiatr Dis Treat 2013;9:1565–71. 24143105 10.2147/NDT.S52311 PMC3797635

